# Recent Progress in the Understanding of the Effect of Sympathetic Nerves on Hair Follicle Growth

**DOI:** 10.3389/fcell.2021.736738

**Published:** 2021-08-26

**Authors:** Jiarui Zhang, Ruosi Chen, Lihong Wen, Zhexiang Fan, Yilong Guo, Zhiqi Hu, Yong Miao

**Affiliations:** Department of Plastic and Aesthetic Surgery, Nanfang Hospital, Southern Medical University, Guangzhou, China

**Keywords:** sympathetic nerves, hair follicles, norepinephrine, hair follicle stem cells, hair growth

## Abstract

Clinical observation and experimental studies have long suggested that the perifollicular nerves have nutritional and regulatory effects on the growth, development, and physiological cycle of hair follicles (HFs), even though the concrete mechanism remains obscure. Recently, with the progress of immunohistochemistry and molecular biology techniques, more innovation has been made in the study of the follicular sympathetic nerves and its nerve-effect factor norepinephrine affecting hair follicle stem cells. This review highlights the progress in the regulation of the sympathetic nervous system toward the growth of HFs.

## Introduction

Immature hair follicles (HFs) are divided into three enclosed epithelial cylinders as highly sensitive, dynamic micro-organs developed from the embryonic epidermis. The largest cylinder in the center forms the hair shaft (fiber), and the outermost cylinder forms the outer root sheath (ORS), which separates the intact structure from the dermis. In the middle, the inner root sheath (IRS) shapes the hair shaft and induces its outward growth (Paus and Cotsarelis, 1999). Mature HFs contain the principal cell types and experience a repetitive growth cycle comprising alternating phases of growth (anagen), degeneration (catagen), and resting (telogen), which are affected by many growth factors, cytokines, hormones, and various neuropeptides (Dry, 1926; Stenn and Paus, 2001; Schneider et al., 2009; Geyfman et al., 2015).

Presenting normally as an indispensable part of the physiological environment of HFs, the sympathetic nerves play an important role in the growth of HFs.

Our review focuses on the biological characteristics of the sympathetic nerves, along with the application of its nerve-effect factor, norepinephrine (NE), in HF growth.

## Sympathetic Nerves Distribution in HFs

HFs undergo various stages of morphogenesis, which is consistent with a certain state of innervation (Paus et al., 1997). From the beginning, the nerve bundles gradually infiltrate the subepidermal interstitial layer and build up an increasingly denser innervation network with the development of the hair cycle. In general, HFs are dominated by the autonomic nervous system, which differentiates into three species: the first one so-called the subepidermal neural-plexus (SEP) stays above the aponeurosis. Under the epidermis there comes with the second layer named subcutaneous neural-plexus (SCP). There are deep dermal neural-plexus (DDP) between the dermis and the developing subcutaneous tissues (Paus et al., 1997). As a branch of the autonomic nervous system all over these aspects, the sympathetic nerves are critical for maintaining body physiology under steady state (Karemaker, 2017). Simultaneously, compact vascular networks synchronize the creating sequence and spatial distribution with the nerves between the epidermis, aponeurosis, dermis, and subcutaneous tissue. It suggests a parallel regulation mechanism in space and time from the beginning of the development of nerves in HFs.

Located between blood vessels and HFs, the sympathetic nerves form a tight connection with the arrector pili muscles (APMs) and vascular smooth muscle. In the dermis, sympathetic nerves converge as a single nerve fiber. Enwrapped and penetrated by sympathetic nerves, APMs provide stable anchors that maintain sympathetic innervations to the HFs. Meanwhile, such a reticular network at the dominant site surrounds the hair follicle stem cells (HFSCs) and forms synapse-like connections with HFSCs. Adjacent to the site where APM inserts into the connective tissue sheath of HFs, sympathetic nerve fibers intermingle but extend beyond the APMs, thus composing a spatial connection with the HFSCs at different positions throughout the outer bulge region and the hair germ (Botchkarev et al., 1997, 1999).

**FIGURE 1:**
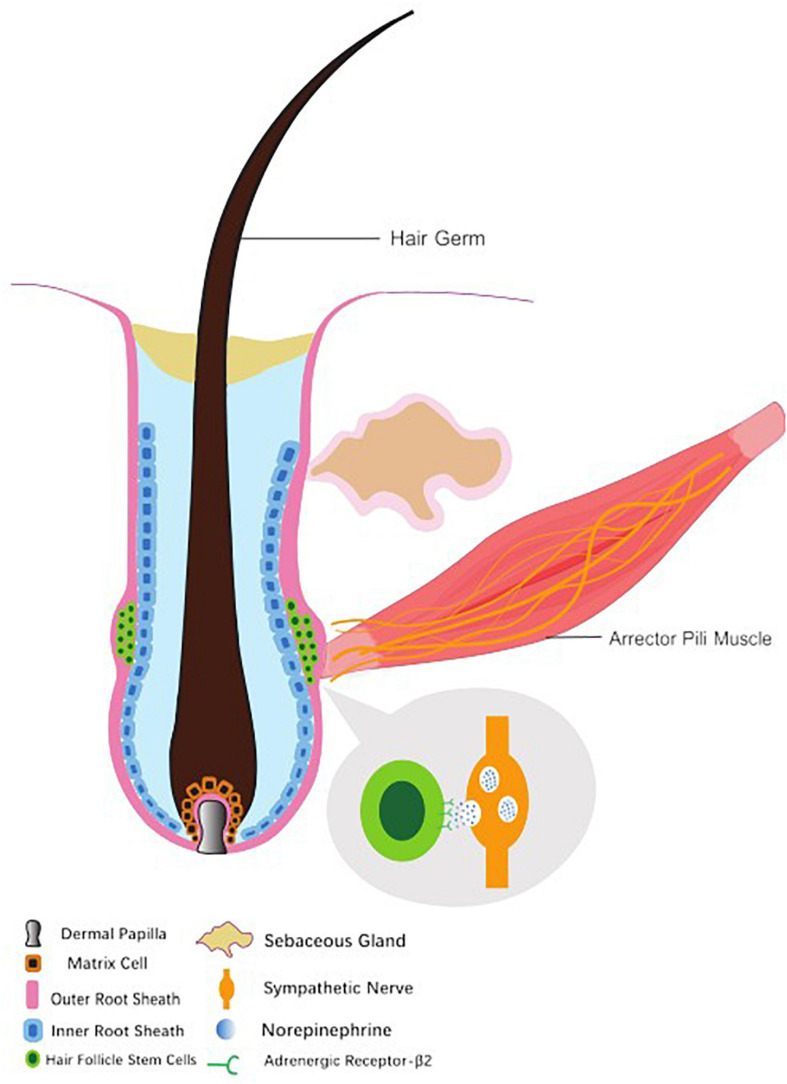
Hair follicle structure, hair follicle stem cell and accessory organs. The arrector pili muscle provides as an anchor to maintain sympathetic innervation to hair. The sympathetic nerves form synapse-like connections with hair follicle stem cells and regulate them through vesicles containing norepinephrine.

In addition, sympathetic fibers are wrapped by the endoneurium composed of specialized collagen and Schwann cells. As the sympathetic nerves approaches HFSCs, the bundled endoneurium opens only on the side that faces HFSCs, exposing nerve fibers to HFSCs, thus preferably enhancing the diffusion of neurotransmitters such as NE-containing vesicles toward HFSCs. This suggests that the sympathetic nerves innervate not only APMs, but also HFSCs in the extra-follicular bulge area and hair germ layer (Shwartz et al., 2020; Figure 1).

## Correlative Changes in the Sympathetic Nerves and Sympathetic Neuropeptides in HFs

Because of the insufficiency of melanocytes in the epidermis, the pigmentation in the trunk of C57BL/6 mice depends completely on the melanin-producing melanocytes in their HFs, whereas the production of melanin in HFs only remains active during their growth period. Also, the color-synchronized growth phases of C57BL/6 mice allow for the isolation and analysis of HFs at specific stages induced by hair extraction (Chase and Eaton, 1959). Therefore, evaluating the skin color conversion of C57BL/6 model macroscopically would be convenient to identify the cycle of HFs—anagen (black), catagen (gray), and telogen (pink) phases. It provides an ideal system for exploring the interaction between sympathetic nerves and the HF cycle.

Considering the characteristics of uniform hair growth waves induced by shaving the back of C57BL/6 mice, Muller-Rover et al. (2001) and Peters et al. (2001) used immunofluorescence and neuronal markers to rearrange the periodic development of dorsal nerves in C57BL/6 mice. Peters found that the general innervation had significant changes and remodeling at specific phases of the hair cycle. The plasticity of HFs innervation in C57BL/6 relied on hair cycle—the amount of autonomic rhythmic nerves amplified significantly during the early anagen, while plummeting in the catagen and reaching the lowest level in the telogen (Steinkraus et al., 1996; Slominski et al., 1998, 2007; Peters et al., 2002). The hair embryo is immunoreactive to most of the neuronal markers. During the period of mouse fetal development at E14/E15 with the mature dorsal root nerve, we could not detect morphologically distinguishable hair embryo formation in the dorsal skin nor the nerve fiber bundles (Steinkraus et al., 1996; Slominski et al., 1998, 2007; Peters et al., 2002).

These findings suggest that the formation and sequential appearance of various types of nerve endings, including sympathetic nerves, are determined by HFs rather than programmatically produced by the dorsal root ganglia (Steinkraus et al., 1996; Slominski et al., 1998, 2007; Peters et al., 2002).

Botchkarev et al. (1999) observed the sympathetic localization of HFs in experimental C57 mice. Compared with the telogen phase, the formation of sympathetic adrenergic and tyrosine hydroxylase (TH) nerve fibers in the dermis and subcutaneous tissue of synchronized HFs increased sharply in the early anagen phase, but decreased steeply in the catagen phase (Botchkarev et al., 1999; Slominski et al., 1999; Kim et al., 2011)—TH antigen is a specific marker of adrenergic nerves (Ljungberg and Johansson, 1993).

Proopiomelanocortin (POMC) is an upstream regulator of the sympathetic nerves in the skin that regulates the secretion of various hormones, including adrenocorticotropic hormone (ACTH), melanocyte-stimulating hormone (MSH), and β-endorphin. The skin not only serves as the target of POMC but also expresses the POMC gene to promote the secretion of related peptides (Paus et al., 1999). The transcription and translation of the POMC gene in the skin of C57BL/6 mice are hair cycle-dependent, which shoot up dramatically in the anagen, but decay in the catagen, and rest in the telogen (Paus et al., 1999). With the application of reversed-phase high- performance liquid chromatography combined with a specific radioimmunoassay, various hair cycle-related sympathetic neuropeptides could be observed in the skin of C57/BL6 mice: the concentration of ACTH was low in the early anagen but was boomed in two steps. The first rapid rise appeared in the early anagen, followed by the second slow climb out in anagen VI. While in the catagen, the concentration of ACTH descended rapidly to the lowest level in the telogen (Slominski et al., 2001).

These changes are accompanied by a parallel expression of adrenergic neurotrophic factor and beta 2-adrenoceptor (β2-AR), -the specific adrenergic receptor on HFs. Likewise, keratinocytes containing β2-AR can be gradually detected in the non-follicular circulating epithelium (especially in the isthmus and bulge region) during the early anagen (Slominski et al., 2001). Moreover, a significant increase in the number of TH-IR nerve fibers accompanied by exocrine norepinephrine secretion was detected by histochemistry (Slominski et al., 2001). During the catagen, they degenerated to a feeble level in the proximal inner root sheath (IRS) and became undetectably in the telogen (Slominski et al., 2001).

In summary, the periodic physiological distribution of sympathetic nerves in HFs and the diversity of adrenoceptor expression show that the growth of sympathetic nerves is related to the growth cycle of HFs. Furthermore, searching for the periodic expression level of sympathetic nerve-related peptides and upstream and downstream regulatory factors in HFs exploit new ideas regarding of the effect of sympathetic nerves on HF growth and the treatment of pathological situations of HFs.

### The Effect of Sympathetic Nerves on HF Stress System

**FIGURE 2:**
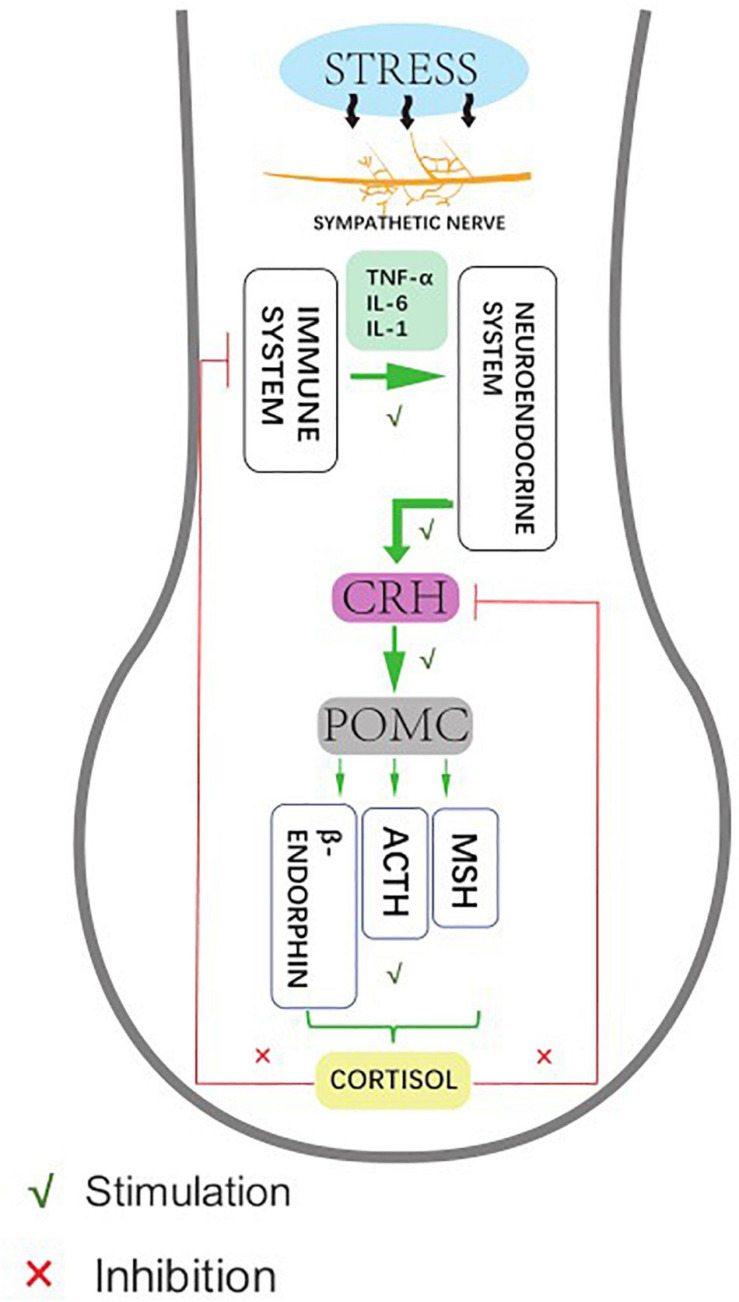
Human hair follicles display HPA axis-like regulatory feedback systems. Affected by hair stress-sensors, the sympathetic nerve activity elevates to different degrees, showing neuroendocrine production and release of corticotropin releasing hormone (CRH), which leads to signal transduction pathway enhances the production and secretion of the anterior pituitary-derived POMC peptides, ACTH, and β-endorphin. Meanwhile, the glucocorticoid receptor agonist cortisol down-regulates follicular CRH expression as an adaptive response to stabilize and restore the general homeostasis.

As a unique and sensitive mini-organ, the HF represents not only the target of stress hormones and autoimmune responses, but can also secrete related stress factors that affect skin conditions (Welch, 1992; Pacak and Palkovits, 2001). The systematic biological response of organisms to external stressors (or classical stress responses) mainly activates the central hypothalamic-pituitary-adrenal (HPA) stress axis, which in turn releases corticotropin-releasing hormone (CRH) to activate the receptor (CRH-R) in the hypothalamus. The downstream target organ-adrenal gland gives out adrenocorticoid-derived peptide (POMC), and the sympathetic nerves produce adrenocorticoid into the blood circulation (Ito et al., 2005). The stress response also comprises autonomic sympathetic regulation of target organs and the immune system: the sympathetic neuropeptides affect the function of the immune system, while the immune system regulates the function of the central nervous system by releasing cytokines. In organ culture and microdissection of HFs, CRH up-regulates the expression of upstream sympathetic molecules, such as POMC, ACTH, and α-MSH peptides. These molecules promote the secretion of cortisol by HFs, which accumulate and exocrine in the keratinocytes of ORS in HFs, and then inhibit the expression of various stressor hormones in ORS. This suggests that HFs have a similar classical HPA axis negative feedback regulation effect (Ito et al., 2005; Figure 2).

Recently studies have shown that a functional stress response system acts independently of the central HPA axis in the HFs, which performs and coordinates the peripheral stress response (Arck et al., 2006; Ito, 2010). Emerging research have suggested that stress is a trigger of common dermatoses—deficiency of the stress system may influence both the HF immune and hormone systems, leading to the induction of autoimmune hair diseases such as alopecia areata (AA) (Alexopoulos and Chrousos, 2016). Psoriasis episodes are often preceded by stressful decreased expression of CRHR-1 which was observed in psoriatic epidermis or dermis, whereas increased levels of CRH were found in the serum of patients with psoriasis. Of note, the development of mast cells in the hair follicle is stimulated by CRH (Basavaraj et al., 2011). Stress also plays a significant role in exacerbating and perpetuating the itch-scratch cycle in atopic dermatitis. Psychological distress stimulates the HPA axis of sympathetic system, which in turn increase secretion of inflammatory mediators that play a significant role in disturbing the skin barrier (Rampton, 2011). What’s more, stress may suppress the activation of the cutaneous HPA axis through glucocorticoids and, thereby, reduce melanogenesis to account for vitiligo (Pang et al., 2014). The sympathetic nerves are stimulated by stressors, which can regulate a variety of HF stress responses by releasing NE, which is the key regulatory point of the HF local stress system (Webster et al., 2002; Botchkarev, 2003; Takenaka et al., 2017). Prospectively, stress caused by the sympathetic nerves around the HFs might be a research focus in dermatology.

### The Effect of Sympathetic Nerves on Hair Follicle Immune System

Early studies have shown that both primary and secondary lymphoid tissues are innervated by postganglionic sympathetic neurons, which secrete NE as primary neurotransmitters, while immune cells express adrenergic receptors, which bind to epinephrine and norepinephrine, enabling the immune system to respond directly to signals from the autonomic nervous system (Nance and Sanders, 2007). When the pattern recognition receptors (PRRs) on the HF sympathetic nerves are stimulated by pathogen-related molecular patterns (PAMPs), the sympathetic neurons suddenly secrete NE.

NE can regulate a variety of immune functions, both systematic and local. The most prominent is the anti-inflammatory effect of NE on congenital and acquired cells. Studies have shown that the adrenergic receptor signals on T cells regulate their transport to surrounding tissues and secondary lymphoid organs (Nakai et al., 2014). They proposed that NE not only inhibits the secretion of inflammatory cytokines and cytolytic activity of mice and human effector CD8^+^ T cells, but also indirectly limits the extent of CD8^+^ T-cell initiation by inhibiting the cross-presentation of dendritic cells (Estrada et al., 2016). In addition, the mechanism of NE-mediated inhibition of tumor necrosis factor-alfa (TNF-α) secretion induced by lipopolysaccharide from pathogenic bacteria involves the junction molecule beta-inhibitory protein, which is regulated by the post-translation of adrenergic signals. The beta-inhibitory protein blocks the activation of immune transcription factor-NFκB (Takenaka et al., 2017). Didem has shown that NE inhibits the secretion of various pro-inflammatory cytokines induced by pathogenic stress and rapidly induces the expression of the anti-inflammatory cytokine IL-10 by specifically binding to ADRB2 (Agac et al., 2018).

## The Effect of Sympathetic Nerves on Hair Follicle Growth

The sympathetic nerves play an important role in the skin nutrition (Agac et al., 2018). Clinically, the experimental subcutaneous injection of the sympathetic chemical toxin 6-hydroxydopamine (6-OHDA) into neonatal mice selectively damaged the terminal nerves containing NE in skin. Subsequently, the morphological interruption of mice HFs was observed. However, isoproterenol, a sympathetic β-adrenoceptor agonist, could partially restore hair growth and even restore HF growth and development (Asada-Kubota, 1995). The premature development of HFs appeared after the induction of over innervation of adrenergic nerves in the denervated skin, which indicates that the sympathetic nerves have an important effect on normal hair growth (Crowe et al., 1993). As the researchers found a substantial reorganization of follicular sympathetic adrenergic cutaneous innervation accompanied by the HF regeneration cycle in mice, great attention was paid on the profound changes in NE secretion (Crowe et al., 1993). After using guanidine to selectively deplete the NE reserve of sympathetic nerves endings, hair growth was inhibited. Then, the cycle does not recover until the adrenaline level is restored (Kong et al., 2015).

**FIGURE 3:**
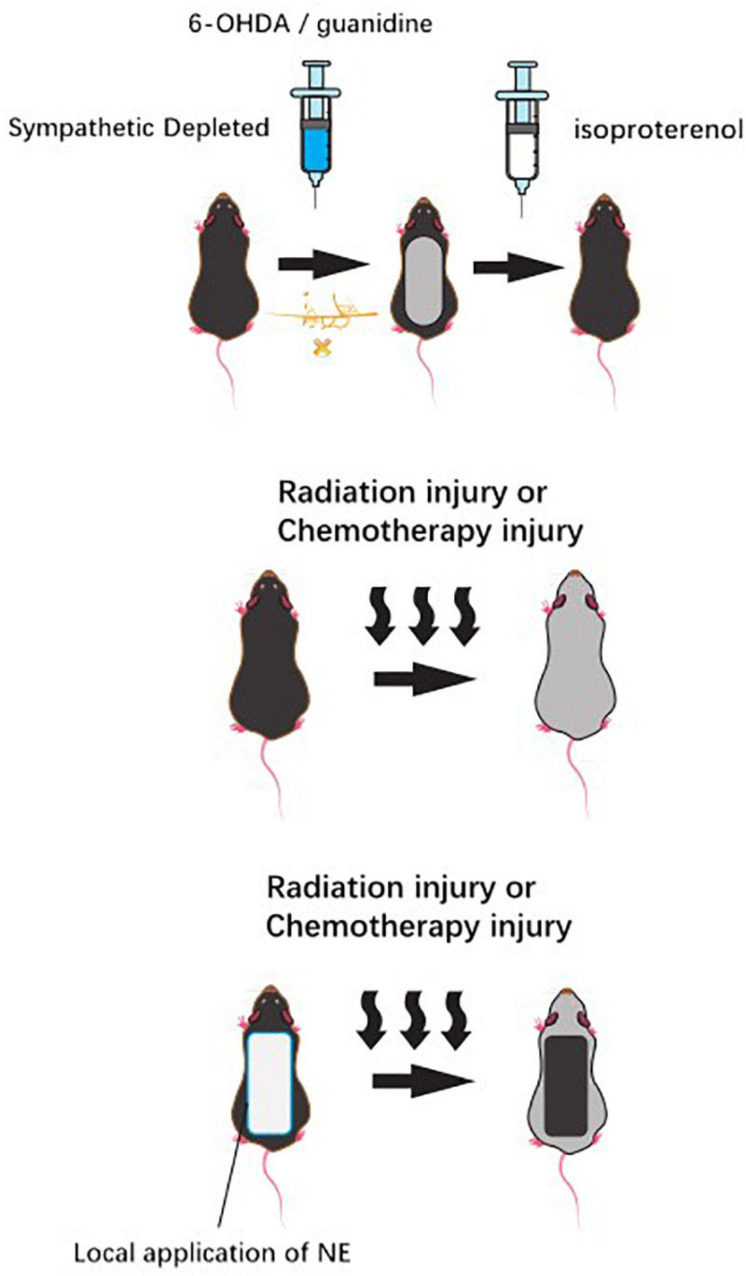
Chemical sympathetic nerve depletion via administration of the neurotoxin 6-hydroxydopamine (6-OHDA) or guanidine to mice inhibited HF growth, while usage of isoproterenol recovered the hair growth. Likely, topical application of norepinephrine to the back skin of rats before a large radiation insult or large systemic chemotherapy insult can prevent the alopecia that was seen in vehicle control animals.

In tumor therapy, the local application of NE skin patch to neonatal rats before high-dose radiation injury or high-dose systemic chemotherapy injury can prevent traumatic alopecia after radiotherapy and chemotherapy (Soref and Fahl, 2015; Figure 3).

This phenomenon not only discusses the general strategy of local application of epinephrine or NE to prevent alopecia caused by cancer treatment, but also efficiently reflects that epinephrine may play an important role in the maintenance of normal hair growth.

Murphy et al. (1998) and Grando et al. (2006) reported that hypodermic injection of epinephrine leads to premature hair growth in the lower back of C57BL/6 mice, suggesting that sympathetic neurotransmitters promote hair growth in a low concentration-dependent manner. The sympathetic nerves innervate into the arrector pili muscles of HFs as part of the HFSC niche and regulates a small portion of the SCs in the upper bulge region by providing ligands for sonic hedgehog (SHH) (Fan et al., 2018; Chen et al., 2020). Phenomena mentioned above state that the sympathetic nerves exert positive influence on hair growth and has considerable research prospects.

Recently, the new concept of the sympathetic regulation of the HF triad has been proposed, where the arrector pili muscles (APMs) and sympathetic nerves form a dual-component niche to modulate hair follicle stem cell (HFSC) activity, which means sympathetic neurons directly regulate stem cells with NE through synaptic-like structures, while the APMs maintain sympathetic innervation of stem cells. Such a tri-lineage unit formed in the skin is stimulated by cold, causing not only sympathetic excitation and contraction of pili muscles, manifesting as goosebumps, but also activates HFSC. In the absence of NE signaling, HFSCs enter deep quiescence by down-regulating the cell cycle and metabolism and up-regulating the resting regulatory factors Foxp1 and Fgf18. With the progression of the hair cycle, descendants of HFSCs secrete SHH to guide the formation of APM sympathetic niches, which control the regeneration of adult HFs (Fan et al., 2018; Chen et al., 2020). The triad system provides a powerful persuasion for the theory of the sympathetic nerves affecting hair growth.

## Side Effects of Sympathetic Nerves Over-Activation on Hair Depigmentation

Researchers have reported that a low concentration of 750 nM NE injected into the back of C57BL/6 mice significantly promotes the proliferation of corneal epithelial cells, while 100 μM NE has a toxic inhibitory effect (Asada-Kubota, 1995; Agac et al., 2018). We suspect that the accumulation of norepinephrine brought by sympathetic nerves over-excitation plays a biological role in a concentration-dependent manner. It might be applied to HF melanocyte stem cells affected by the norepinephrine accumulation, leading to the fallout of hair pigmentation.

Related to the clinically observed phenomenon called “overnight white-head,” which means the rapid destruction of hair pigmentation, a study indicated that acute stress leads to hair graying through the rapid depletion of melanocyte stem cells. Loss of pigmentation was independent of immune attack or adrenal stress hormones but resulted from activation of the sympathetic nerves, which changes the melanocyte niche. The motionless melanocyte stem cells proliferate and differentiate rapidly, then migrate outward from the normal survival area, and become permanently exhausted, causing irreversible whitening of black hair (Zhang et al., 2020).

Abnormal sympathetic nerves distribution was observed in the skin lesions of patients with alopecia areata (Tatu et al., 2019). In this regard, we assume that abnormalities in sympathetic nerves distribution or morphology might follow androgenetic alopecia (AGA) or senile alopecia, both of which are worthy of further study.

## Discussion

A large amount of experimental data has confirmed the reciprocal communication between the sympathetic nerves and HFs, which provides novel insights into the mechanisms of physiology progression on hair growth. Shwartz et al. discovered that the sympathetic nerves secreted NE to activate hair growth of mice (Tatu et al., 2019), and Zhang proposed the stress hormone corticosterone regulates hair follicle stem cell (HFSC) quiescence and hair growth in mice (Choi et al., 2021). At present, it has not been established whether neurogenesis and cross-talk of neuropeptides are coincidental or prerequisites in the development of human hair growth. Moreover, the anomaly of either the characteristic configuration or the distribution of the sympathetic nerves is unknown for incurring the specific dysfunction of HFs in different hair diseases such as androgen alopecia, folliculitis, or senile alopecia. Studies have described the phenomenon of the sympathetic nerves directly activate HFSCs with NE to proliferate in mice. However, the mechanism of the sympathetic nerves affecting hair growth of human is not clear in clinical studies. Hence, the promising correlation of the sympathetic nerves and human HFs cannot be ignored and further investigations are recommended.

The action of HFSCs is controlled by its niche. This microenvironment incorporates the daughter cells of the HFSCs in the bulge, which enact their self-recovery ahead of schedule and in the late anagen stages (Tanimura et al., 2011). Maintaining the niche of HFSCs is vital for HF homeostasis and harm fix. Divisions of HFSCs are not frequent in mature HFs, and their greater part are in a lethargic state. As such, it is vital to comprehend the components of their activation and induction, which will allow for the use of multipotent growth factors in hair regrowth. Their use is complicated by the fact that the expression of receptors on the various growth factors and the effect of the microenvironment may vary. Not all target points in HFSCs therapy have been distinguished (Gentile and Garcovich, 2019).

Without the sympathetic signaling, HFSCs enter deep quiescence by down-regulating the cell cycle and metabolism while up-regulating quiescence regulators Foxp1 and Fgf18. During development, HFSCs progeny secretes Sonic Hedgehog (SHH) to direct the formation of this APM-sympathetic nerve niche, which in turn controls HFs regeneration (Tatu et al., 2019).

Hair growth was also regulated prevalently by the Wnt biomolecular pathway and ERK activation in which HFSCs and growth factors are involved. Specifically, it has been additionally demonstrated that Wnt/β-catenin signaling is necessary for the growth and upkeep of the mesenchymal stem cells (MSCs) (Huelsken et al., 2001; Tsai et al., 2014). The survival and growth of MSCs relies on signs transmitted by the HFSCs, for instance, the TGF-β or the Wnt pathway (Huelsken et al., 2001; Tsai et al., 2014). Likewise, HFSCs are influenced by MSCs in the dermal papilla, which are in close contact with the germinal matrix that are isolated by the basal membrane. They appear to be part of vital significance in the activation of hair growth and in signal transmission during recovery (Rompolas and Greco, 2014). Specifically, Gentile innovatively obtained autologous micrografts containing MSCs from centrifugation of a punch biopsy of the scalp, and used mesotherapy gun accessible for mechanical and controlled MSCs infiltration to treat the affected area of the patients with AGA (Gentile et al., 2020). It is considerable whether the norepinephrine combined with stem cell therapy could better play the synergistic effect of hair growth promotion.

If the sympathetic nerves support hair growth, sympathetic neuropeptides must be secreted at a certain level to maintain the stability of the internal environment of HFs. However, the understanding of the aspect of sympathetic neuropeptides in both stable physiological state and pathological conditions of HFs is still limited.

Recently, the use of autologous platelet-rich plasma (PRP) was determined to provide substantial help in hair regeneration owing to the platelets’ ability to advance neo-angiogenesis, cell expansion, and separation (Cervelli et al., 2009, 2013). Platelet-rich plasma (PRP) are rich in growth factors, of which are engaged in hair bio-molecular activity (Cervelli et al., 2012). DPCs have shown improved proliferation, improved Bcl-2 and FGF-7 levels, activated ERK and Akt proteins, and up-regulation of β-catenin when cultured in an initiated PRP-enhanced growth medium (Li et al., 2012). Each of these elements decidedly impacts hair growth. However, there are too many protocols for the preparation of PRP depending on the different times for centrifugation and RPM used, the number of platelets, the accessibility of growth factors, and chemokines. Thus, it is hard to evaluate which kind of PRP planning is better for clinical indications (Dhurat and Sukesh, 2014). Compared with the PRP, the sympathetic nerves may exert the parallel positive influence on hair regeneration by norepinephrine. The current knowledge in biology, the limits of past translational research will provide a strong basis to advance viable clinical approaches for regenerative aims in hair tissue engineering. Larger, randomized, double-blinded, controlled trials are needed to optimize cell administration protocols and to confirm the early observations of promising clinical outcomes.

Considering the short half-life of neuropeptides and the fast regulation of sympathetic nerves, local application of related drugs may reduce the side effects associated with systemic administration. In recent years, drug carriers such as liposomes, exosomes, and extracellular vesicles (EVs), have been used to effectively deliver neuropeptides into the skin. In a clinical model review, micro-needling is a minimally invasive dermatological procedure in which fine needles are rolled over the skin to puncture the stratum corneum. This therapy is used to induce collagen formation, neovascularization and growth factor production of treated areas. Micro-needling has been used in a wide range of dermatologic conditions, including AGA and alopecia areata (Fertig et al., 2018). Neuropeptides from sympathetic nerves are easily accessible in clinical institution, which facilitates relevant experiments combined with micro-needling on hair growth or wound healing. More exploration of the precise cellular and molecular transmission of the sympathetic neuropeptides in the circumstances of inflammation, dysplasia and atrophy in HFs opens up breaking ground.

Thus, given these factors, the concrete mechanism of sympathetic nerves taking effect on hair growth will be key to elucidating the complex regulation of HFs, and will facilitate the identification of druggable molecular targets for hair disease intervention. It makes sense for further experimental or clinical studies to investigate the translational value or clinical applications of the sympathetic nerves in hair follicles.

## Author Contributions

JZ was responsible for manuscript writing. RC, LW, ZF, and YG were responsible for manuscript modifying. YM was responsible for the conception and design. ZH was responsible for the final approval of the manuscript. RC was responsible for the figure preparation and manuscript modifying. All authors read and approved the final manuscript.

## Conflict of Interest

The authors declare that the research was conducted in the absence of any commercial or financial relationships that could be construed as a potential conflict of interest.

## Publisher’s Note

All claims expressed in this article are solely those of the authors and do not necessarily represent those of their affiliated organizations, or those of the publisher, the editors and the reviewers. Any product that may be evaluated in this article, or claim that may be made by its manufacturer, is not guaranteed or endorsed by the publisher.

## Abbreviations

HF: hair follicle
ORS: outer root sheath
IRS: inner root sheath
APM: erector muscle
β2-AR/ADRB2: β2-adrenocorticoid receptor
POMC: proopiomelanocortin
ACTH: adreno-cortico-tropic-hormone
HPA: hypothalamic-pituitary-adrenal stress axis
AA: alopecia areata
NE/NA: norepinephrine/noradrenaline.
